# Morphometry-based design and finite element assessment of primary stability in glenoid baseplate fixation for reverse shoulder arthroplasty for the Indian population

**DOI:** 10.3389/fbioe.2026.1927447

**Published:** 2026-10-05

**Authors:** Pearline Beulah John, Sharmila Nageswaran

**Affiliations:** School of Electronics Engineering, Vellore Institute of Technology, Vellore, India

**Keywords:** finite element analysis, glenoid baseplate, glenoid morphometry, indian glenoid, reverse shoulder arthroplasty

## Abstract

**Introduction:**

The geometrical and morphological parameters of the glenoid vary across ethnic groups, affecting the fixation of the baseplates in reverse shoulder arthroplasty. Studies have shown that using larger baseplates for smaller glenoids may not facilitate a better biomechanical outcome, and therefore a population-based investigation plays a crucial role in selecting an appropriate implant.

**Methods:**

This work investigates finite element analysis of a pear-shaped glenoid baseplate, designed based on the morphometric parameters of the Indian glenoid. A percentile-based method was used to select the representative glenoid to select small, middle and large glenoid variations within the Indian population. Nine glenoid-baseplate assemblies were assembled from three different glenoid dimensions and three baseplate sizes. This baseplate-scapula construct was subjected to finite element analysis under specified loading conditions for various abduction angles to investigate mechanical load sharing and primary stability.

**Results:**

These findings indicated that the fixation outcomes changed based on the baseplate-glenoid assembly, showing that the biomechanical performance did not depend on the baseplate size alone.

**Discussion:**

Smaller baseplates and intermediate baseplates showed better primary stability in the Indian glenoid, supporting morphometry-guided baseplate selection based on implant-glenoid dimensional compatibility.

## 1 Introduction

Reverse total shoulder Arthroplasty is a widely used treatment for massive cuff tear arthropathy, proximal humeral fractures, revision surgeries, and post-operative complications or failed total shoulder arthroplasty. Regardless of the developments in implant architecture, challenges arise due to instability, scapular notching and glenoid loosening, contributing to revision surgical procedures (Boileau, 2016; Wright et al., 2015). However, limitations in the existing design have accelerated the need for revision surgeries. Some of the recurrent complications include glenoid loosening, scapular notching, infection, deltoid injury and factors affecting the stability of the joint. High rates of complication were associated with component loosening and instability of the joint, which is about 12.4% and 5%, respectively (Bohsali et al., 2017). With regards to component loosening, glenoid base plate loosening is the most common problem that arises due to poor stability of the joint, inferior quality of the bone and deficient porous ingrowth on the implant (Diaz et al., 2020).

Implant dimensions are crucial in restoring the joint range of motion (ROM) and providing an optimal fit. Small changes, such as dimensions of about 5mm, can alter the functionality of the articulating joint (Sahu et al., 2020). Shoulder prosthesis that are available in the market have been designed in accordance with the morphology of the Caucasian population. It was found that there was a geometric mismatch between the bone and the implants used for total knee arthroplasty due to the morphological changes observed between the bones of Asians and the Western population (Sahu et al., 2020). Consequently, geometric measurements of the bones of the glenohumeral joint and variation in dimensions between the Western population and Indians were ascertained by Meshram et al. (2020), and Sahu et al. (2020), as it can assist in the development of shoulder implants for Indians.

The discrepancy between the height and width of the glenoid creates uncertainty in selecting the base plate that matches the glenoid (Meshram et al., 2020; Amy et al., 2016). Geometric measurements of the glenoid for the Indian population are found to vary for different individuals belonging to various races based on regional diversity. Despite these changes, the mean glenoid measurement is found to be around 23–24 mm based on the demographic dissimilarities. It is documented that in 68% of the population, the glenoid width was less than 25 mm (Sahu et al., 2020). Nevertheless, the smallest baseplate diameter, commonly known as the mini baseplate, available in the market is about 25 mm. Commercially available glenoid base plates range from 25 mm to 34 mm. Comparative studies between a 25mm and 29 mm base plate were carried out, and it was found to improve implant stability, low micromotion and a wide ROM without impingement (Chae et al., 2014; Athwal and Faber, 2016). Table 1 provides a comparison of the dimensions of the glenoid in the Indian and Western populations.

**TABLE 1:** Glenoid dimensional differences between Indians and Western population.

Glenoid dimensions	Indians (mean ± SD)	Western (mean ± SD)
Glenoid width (mm)	24.0 ± 2	29 ± 3.1
Glenoid SI length (mm)	31.3 ± 2.2	39 ± 3.7

Fixation and stability of the glenoid baseplate are significant functional biomechanical requirements for avoiding post–surgical complications and enhancing implant longevity. The primary stability of the glenoid baseplate is dependent upon the implant design, its fixation, screw configuration, central screw fixation and the density of the scapular bone (Bonnevialle et al., 2020; Torkan et al., 2022; Spek et al., 2024). In addition, the primary stability of the implant is dependent on the bone-implant interfacial contact, where increased interfacial motion can hinder initial bone formation potential. Micromotion at the bone-prosthesis interface is commonly used to evaluate the primary stability, where a relative motion of less than 150 µm is considered beneficial for osteointegration and bone remodelling (Bonnevialle et al., 2020; Formaini et al., 2015).

The difference in anatomy has a wide impact on the biomechanical and surgical significance. A large baseplate can create overhang in the periphery, increased glenoid reaming, insufficient screw engagement with the surrounding bone and modified load distribution. A finite element study on 29 mm and 25 mm baseplates suggests that the primary fixation stability of the baseplate size is dependent on the morphology of the glenoid. The 25 mm baseplate indicated enhanced primary fixation when compared with a 29 mm baseplate, demonstrating that in small glenoids, a geometrical match can improve bone-screw contact and available bone stock for peripheral screw fixation (Kim and Chae, 2026). Hence, it is imperative to determine baseplate sizing depending on the geometrical and morphological parameters rather than implementing generalised implant dimensions across various populations.

FEA and biomechanical studies have been useful in determining the bone-baseplate interaction, micromotion, screw fixation and the impact of bone loss on primary stability and fixation (Bonnevialle et al., 2020; Torkan et al., 2022; Spek et al., 2024; Formaini et al., 2015; Kim and Chae, 2026). Shoulder force measurements have indicated that glenohumeral contact loads can change depending on the position of the arm during elevation, thereby reinforcing the application of abduction-based loading for assessing shoulder implant fixation (Bergmann et al., 2007). Glenoid loosening contributes to osteopenia and loss of bone.

The glenoid cavity is a complex structure showing variations in its external and internal osseous morphology. The differences in the shape of the glenoid cavity have been related to the contour of the articular surface and the morphology of the anterior glenoid notch. Prescher and Klumpen noted that a pear-shaped glenoid cavity was associated with the glenoid notch, while an oval-shaped cavity was associated with the glenoid notch being absent (Prescher and Klümpen, 1997). Checroun et al., in an anatomical assessment of 412 glenoids, classified 71% as pear-shaped and 29% as elliptical (Checroun et al., 2002). Recently, Chen et al., using 3D reconstruction, classified the glenoid cavity into five configurations, namely, the pear-shaped, oval-shaped, teardrop-shaped, calabash-shaped and inverted-comma-shaped (Chen et al., 2023). The anterior glenoid notch represents another feature that has been classified according to its depth as mild (Type I), moderate (Type II) or severe (Type III) (Alashkham et al., 2017). Several anatomical findings have also reported inconsistencies in the terms used to describe the structures of the scapula. Al-Redouan and Kachlik put forward sixteen terms for grossly visible scapular features through Delphi consensus and expert taxonomic review (Al-Redouan and Kachlik, 2022). Benes et al. distinguished two osseous variants within the glenoid fossa as the intraglenoid tubercle and glenoid fovea and defined standardised terminology for the associated articular-surface variations (Benes et al., 2023). The articular cartilage that overlies these bony variants was found to be thinner, and in some young individuals, the cartilage can be absent over the glenoid fovea. These indications reveal that the glenoid morphology has different regional variations, and these features can add relevance during the glenoid cavity preparation and implant fixation (Benes et al., 2023). These observations indicate that glenoid morphology includes regional anatomical variations in addition to overall linear dimensions.

The glenoid rim and the bone stock available at the periphery around the baseplate footprint are significant to primary fixation. In small glenoids, large baseplates can reduce the amount of bone available for peripheral screw fixation; however, small baseplates have been shown to lower micromotion and improve screw support when matched with the available glenoid dimensions (Chae et al., 2014). Recent FEA analysis has demonstrated that a smaller baseplate in a small glenoid offered better cortical rim engagement, increased bone-screw contact, greater length of supporting bone stock and decreased micromotion (Kim and Chae, 2026). Physiological thinning of the glenoid articular cartilage has also been associated with underlying osseous variations, specifically the intraglenoid tubercle and glenoid fovea, which can be of importance during glenoid preparation and fixation of the baseplate (Benes et al., 2023). These anatomical considerations offer relevant context for evaluating baseplate fixation in relation to glenoid dimensions.

The recent developments in glenoid baseplate design in reverse shoulder arthroplasty comprise different designs based upon component diameter, lateralization, augmentation and patient-specific geometry tailored to address variations in glenoid size, bone stock and deformity. In Asians with a relatively small glenoids, Kim et al. found that a smaller 29.5-mm baseplate reduced glenoid–baseplate size mismatch and glenoid vault perforation compared with a standard 33.8-mm baseplate, demonstrating the importance of component sizing and the dimensions of the glenoid (Kim et al., 2023). Lateralized baseplates have been associated with improved short-term patient-reported outcomes and reduced scapular notching (Baumgarten and Max, 2024), whereas isolated glenosphere lateralization has also been associated with reduced scapular notching (King et al., 2023). Augmented baseplates have shown favourable clinical results for glenoids with substantial glenoid version or inclination deformity (Levin et al., 2022; Colasanti et al., 2023). CAD/CAM-based patient-specific baseplates have been implemented in conditions of severe deformity or bone deficiency (Rashid et al., 2023; Moran et al., 2024; Bodendorfer et al., 2021). Despite these advances, population-specific results linking glenoid morphometry with appropriate baseplate size are limited.

Various anatomical studies from different regions of India have assessed the distribution of the morphological configuration of the glenoid. In the North Indian population, pear-shaped glenoids were reported in 48.78% of right and 46.15% of left scapulae (Dhindsa and Singh, 2014). Another Indian study reported pear-shaped, inverted-comma-shaped and oval configurations in 50.44%, 35.96% and 13.60% of specimens, respectively (Akhtar et al., 2016). In a South Indian cohort, pear-shaped glenoids accounted for 54% of the specimens (Raaj et al., 2018), whereas another North Indian study reported pear, oval and inverted-comma configurations 50%, 29.65% and 20.35% respectively (Singh, 2020). A central Indian study reported corresponding prevalences of 50.66%, 33.3% and 16% (Waha et al., 2021). Altogether, these studies report that the pear-shaped glenoid is a common and predominant morphological configuration in the Indian population. Variations in glenoid contour among pear-shaped, oval and inverted-comma-shaped glenoids can modify the available surface for baseplate seating and the peripheral bone available for fixation, thereby influencing glenoid–baseplate compatibility and primary stability. Commercially available glenoid baseplates are provided in different sizes and design configurations to accommodate variations in glenoid anatomy and fixation requirements, with representative baseplate dimensions listed in Table 2. Accordingly, based on the commonly reported pear-shaped glenoid morphology in the Indian cohorts, a pear-shaped baseplate geometry was adopted in the present study, with different baseplate dimensions evaluated in relation to representative glenoid sizes.

**TABLE 2:** Prosthesis for RSA and the size of the base plate (Meshram et al. (2020).

Company	Prosthesis	Base plate size
Wright, Memphis, United StatesA	Aequalis reversed II	25 mm
DJO surgical, austin, United States	Reverse shoulder prosthesis system	26 mm
DePuy Synthes, Warsaw, United States	Delta xtend system	27 mm
Stryker, New Jersey, United States	ReUnion system	28 mm
Zimmer, Warsaw, IN, United States	Trabecular metal reverse shoulder system	28 mm

Although studies regarding the Indian glenoid morphometric analysis have been addressed in the literature, to the best of our understanding, no study has systematically evaluated the biomechanical compatibility of multiple baseplate sizes across representative Indian glenoid dimensions. The specific contribution of the present study is the systematic evaluation of dimensional compatibility between three representative glenoid sizes and three baseplate sizes, resulting in nine glenoid–baseplate configurations. In this study, representative scapula models were chosen based on geometric parameters of the glenoid, and glenoid baseplates were designed and evaluated for baseplate fixation under abduction loading. Scapulae, which correspond to the 10th, 50th and 90th percentile glenoid width, were chosen for analysis. Finite element analysis was performed to test the primary stability of the implant by determining micromotion, von Mises stress of the implant and the bone, and total deformation. Material properties of Ti-6Al-4V were applied, and a comparative analysis of the primary stability was performed for 0°, 30°, and 60° abduction loading. By incorporating representative Indian glenoid dimensions and finite element-based stability analysis, this work facilitates a population–specific biomechanical basis for glenoid baseplate design and selection for reverse shoulder arthroplasty.

## 2 Materials and methods

### 2.1 3D reconstruction of the scapula bone

50 de-identified, retrospective shoulder computerised tomography DICOM images were obtained from the hospital. 3D Slicer 5.8.0 was used for thresholding, segmentation and reconstruction of the scapular models as shown in Figure 1. The final scapular models were exported in STL format to Fusion 360. DICOM parameters of the representative CT shoulder image are shown in Table 3.

**FIGURE 1:**
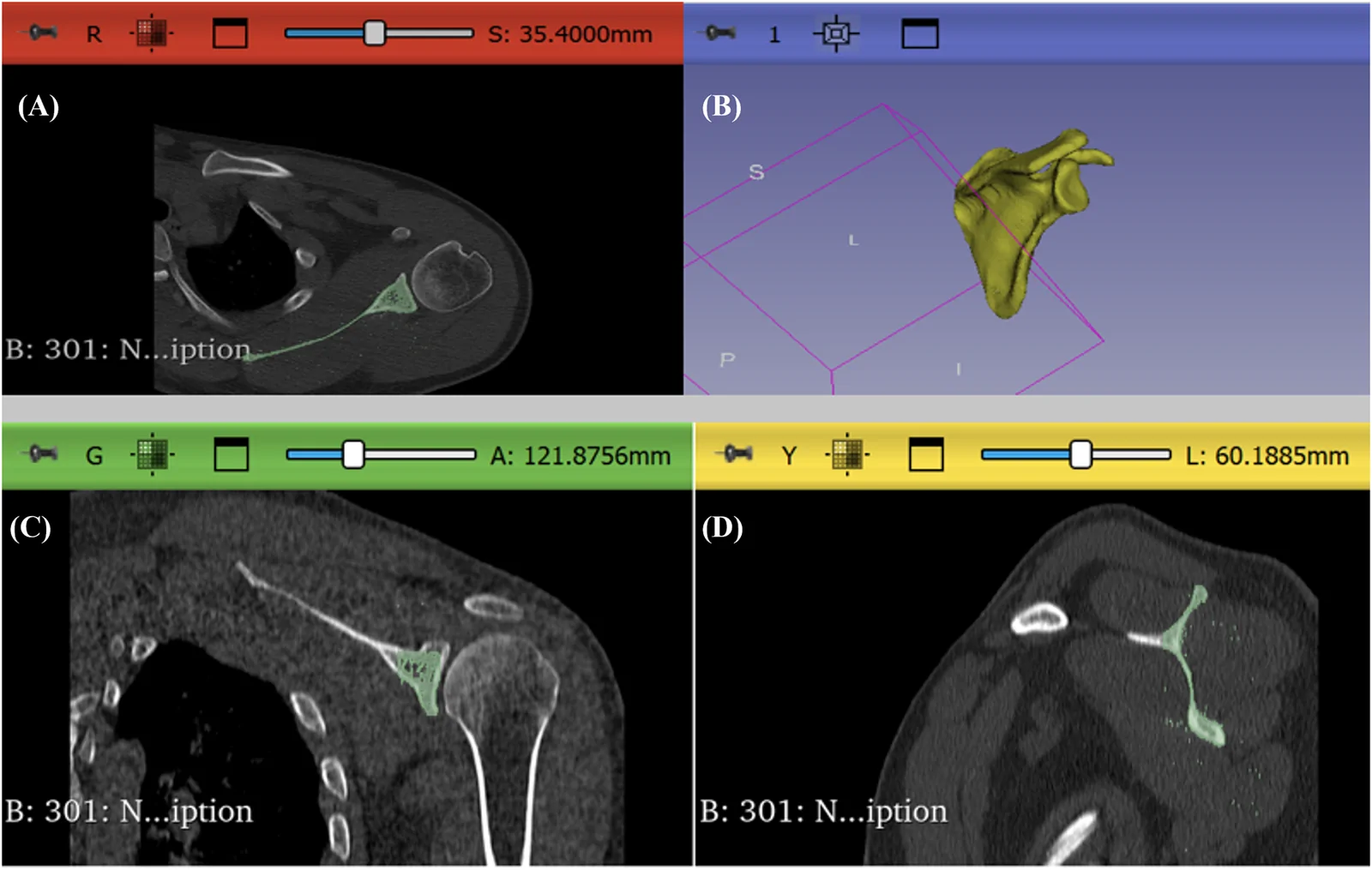
3D reconstruction of the scapula: **(A)** axial CT view, **(B)** 3D reconstructed scapular model, **(C)** coronal CT view, and **(D)** sagittal CT view.

**TABLE 3:** DICOM parameters of the representative scapula.

DICOM parameters	
Matrix size	512 × 512 pixels
Pixel size	0.6124 mm
Slice thickness	1.0 mm
Slice increment	1.0 mm

Ethical clearance was obtained from Sri Narayani Hospital & Research Centre Institutional Ethics Committee, Vellore, India (SNHRCEC/84/30/08/2025), and Institutional Ethical Committee for Human Studies, Vellore Institute of Technology, Vellore, India (VIT/IECH/2025/17IECH/5 JULY2025/16) for conducting the study..

### 2.2 Percentile-based selection of scapular models and statistical analysis

Measurements of the 50 reconstructed scapular models were obtained by a single observer using a standardized measurement protocol in 3D Slicer. Subsequently, three representative scapulae were selected based on the anterior–posterior (AP) width of the glenoid. The measurements were summarized using descriptive statistics, including the mean, standard deviation, and percentile distribution. Scapular models were selected based on the anterior–posterior (AP) width of the glenoid, with the 10th, 50th, and 90th percentiles representing small, median, and large glenoid dimensions, respectively, as shown in Figure 2. The percentile-based method of scapular selection was followed to determine the baseplate that has the potential to match among anatomically relevant sizes rather than selecting an average scapula model. Table 4 shows the descriptive statistics of the glenoid measurements and the geometric dimensions of the glenoid corresponding to the 10th, 50th, and 90th percentiles.

**FIGURE 2:**
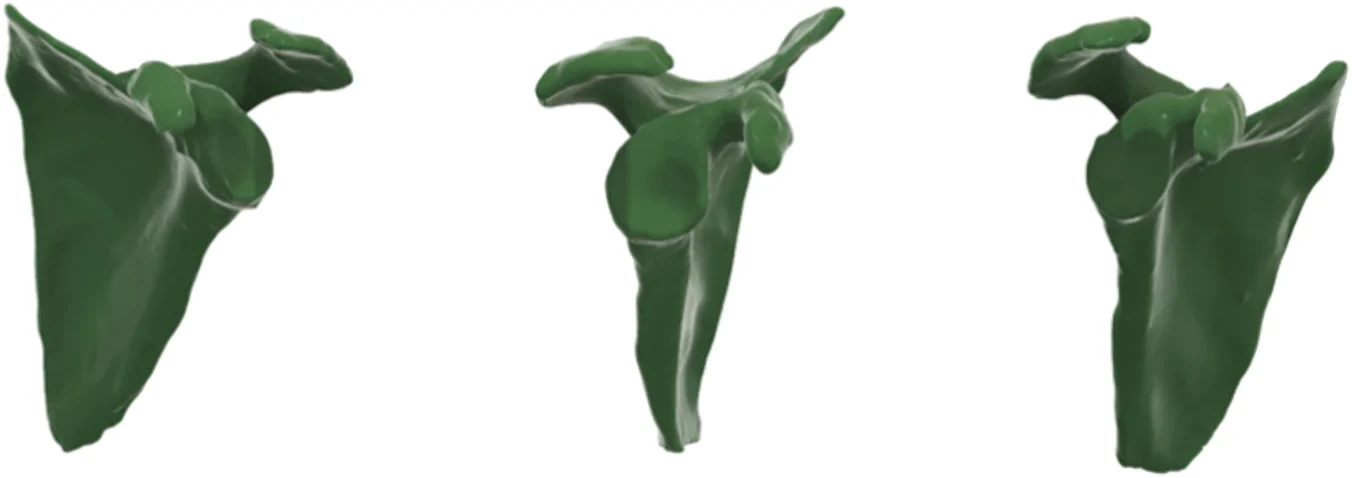
Scapular selection based on the width of the glenoid showing 20 mm, 22 mm and 25 mm glenoid.

**TABLE 4:** Dimensions of the glenoid related to 10th,50th and 90th percentile.

Parameter	Mean ± SD (mm)	Min–Max (mm)	P10 - based scapula (mm)	P50 - based scapula (mm)	P90 - based scapula (mm)
SI length (glenoid height)	33.6 ± 2.4	29.03–39.05	29.65	30.48	33
AP width 1	22.8 ± 2.21	19–27.9	20.3	22.7	25.6
AP width 2	18 ± 2.5	13.5–21.93	15.83	18.97	18.8

### 2.3 Baseplate design, preliminary screening and screw geometry

The reverse shoulder implant for Indian conditions was designed using Fusion 360 software using materials, 316 L stainless steel and Ti-6Al-4V. Finite Element Analysis (FEA) was performed on the designs using ANSYS Workbench 2023, and a compressive load of about 750 N was applied to test the Reverse shoulder implants according to ASTM F2028, which is the standard test method for dynamic evaluation, loosening or dissociation of the implant (Harman et al., 2005).

The glenoid baseplate is placed after the portion of the affected glenoid is reamed out. The Superior- Inferior diameter (SI) and Anterior-Posterior glenoid diameter (AP) are the two crucial parameters that are determined in the measurement of the dimensions of the glenoid. SI diameter is the maximum distance between the prominent part of the supra-glenoid tubercle and the inferior point of the glenoid margin; AP diameter is the width of the glenoid, which can be observed across two regions in Figure 3. Fusion 360 was used to design the baseplate according to the measurements of the Indian glenoid. The glenoid baseplate that has been designed is pear-shaped with holes and a cage, which facilitates the baseplate to firmly fix to the glenoid cavity, with the help of screws. The SI diameter of the glenoid baseplate is 31.3 mm and the AP diameters 1 and 2 are 16 mm and 22.6 mm, respectively, as seen in Figure 3, which was designed initially. Circular, oval and elliptical holes were designed on the area of the baseplate other than those that are used to fix screws to enhance implant bonding and reduce stress shielding, as seen in Figure 3.

**FIGURE 3:**
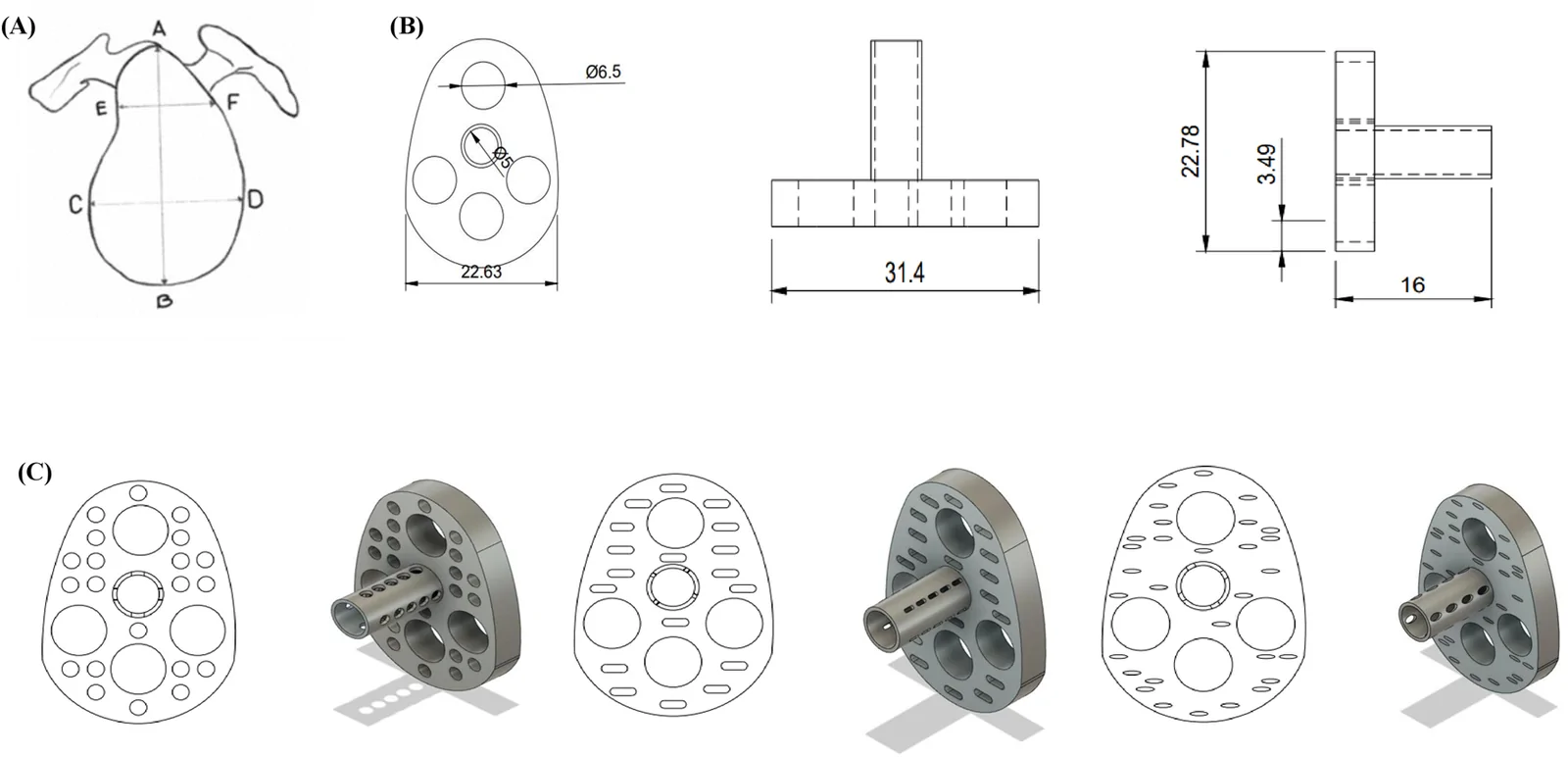
**(A)** A to B → SI Diameter; C to D → AP Diameter 1; E to F→ AP Diameter 2, **(B)** design of the glenoid baseplate, **(C)** base plate designs and 3D models with varying hole patterns.

The baseplate diameter, thickness, central geometry and screw-hole pattern remained the same for all three designs. An initial finite element analysis was conducted on all these models to compare the stress responses of the baseplates. Forces ranging from 500N to 3000 N were applied to the three baseplates for analysis. The load was applied on the proximal part of the base plate, and the restraint was fixed on the cage and the base of the cage. In addition, a comparative study on von Mises stress using 316LSS and Ti-6Al-4V was carried out to assess material selection for analysis.

Four screws, as shown in Figure 4, were designed with an overall length of 15mm, a shaft diameter of 3.3 mm and a head diameter of 7 mm. The same screw geometry was used across all the models. Material properties of Ti-6Al-4V were assigned to the screws.

**FIGURE 4:**
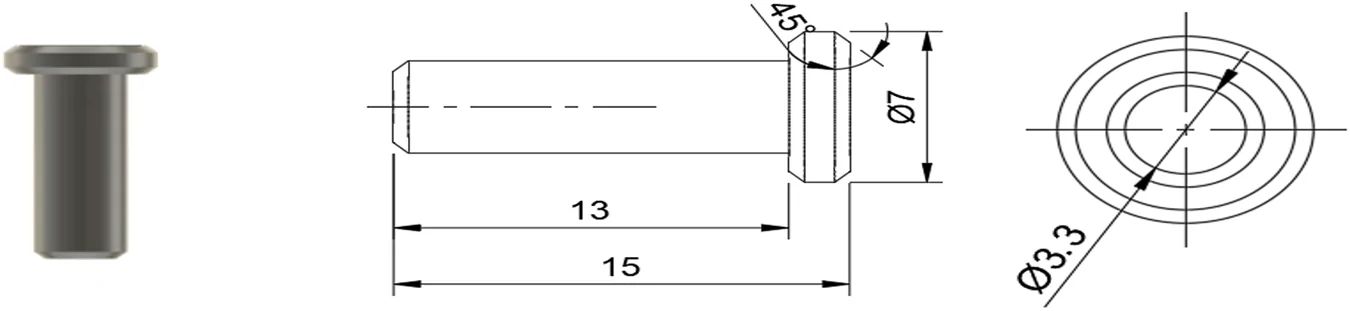
Screw dimensions.

### 2.4 Assembly and finite element model

Three baseplates with diameters of 20mm, 22mm, and 25mm that correspond to the small, median, and large glenoid were redesigned from the selected circular-pattern baseplate models. Using Fusion 360, the glenoid surface was virtually reamed to seat the baseplate to mimic the surgical placement of the implant on the glenoid and to create contact between the implant and the scapular bone, as seen in Figure 5. Four screws were inserted through holes into the scapular bone stock to complete the assembly construct.

**FIGURE 5:**
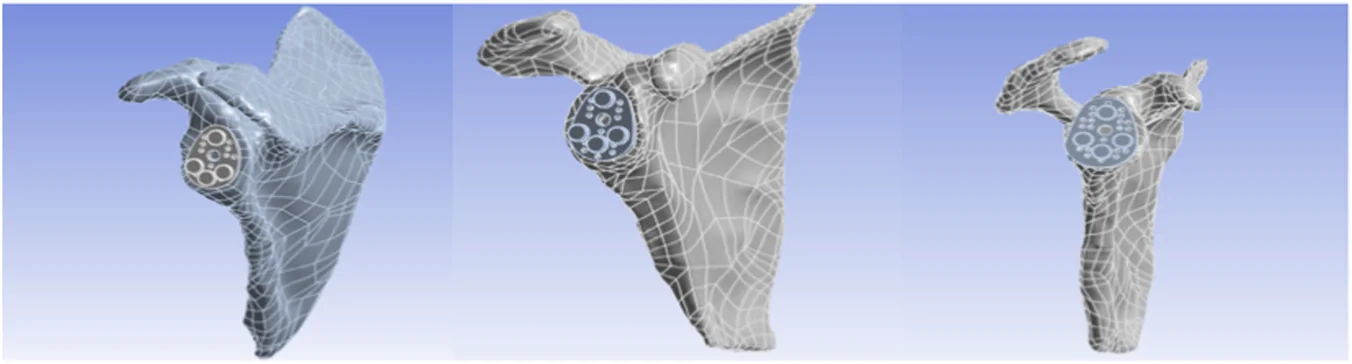
Assembled baseplates and glenoids.

Nine scapula-baseplate assemblies were imported from Fusion 360 to ANSYS Workbench 2023 for finite element analysis. The material properties of Ti6Al4V and bone were assigned in Ansys Workbench 2023. The scapular bone was considered an isotropic, homogeneous, and linearly elastic material. These assumptions were applied to maintain uniformity between various scapular models, materials, baseplate sizes and loading scenarios. Nine scapular assemblies were evaluated for 0°, 30°, and 60° abduction loading conditions. Hence, 27 finite element simulations were carried out. The FEA assembly models were discretised using three-dimensional tetrahedral solid elements with a global mesh setting. Similarly, the same meshing condition was applied to all the models to maintain consistency.

### 2.5 Mesh sensitivity analysis

Mesh sensitivity analysis was carried out by changing the global mesh size and determining the related change in total deformation of the scapula-baseplate-screw assembly. Total deformation was considered as the response parameter as it denotes the overall structural response of the model under loading. Mesh sensitivity was considered acceptable when the change in total deformation between successive refinements was less than 5%. The deformation behaviour showed greater changes in coarser meshes. The largest change was observed between 1.75 mm and 1.5 mm with a variation of approximately 3.4%. This change was below the 5% variation threshold. From 1.5 mm, the deformation response stabilised for 1.5 mm, 1.25 mm and 1 mm meshes with values 0.936 mm, 0.939 mm, and 0.936 mm, respectively, as shown in Figure 6. Hence, a global element size of 1.5 mm was chosen as it ensured numerical stability without incurring high computational cost. Therefore, a 1.5 mm mesh was applied throughout all the baseplate–glenoid assembly models to ensure consistency in analysis.

**FIGURE 6:**
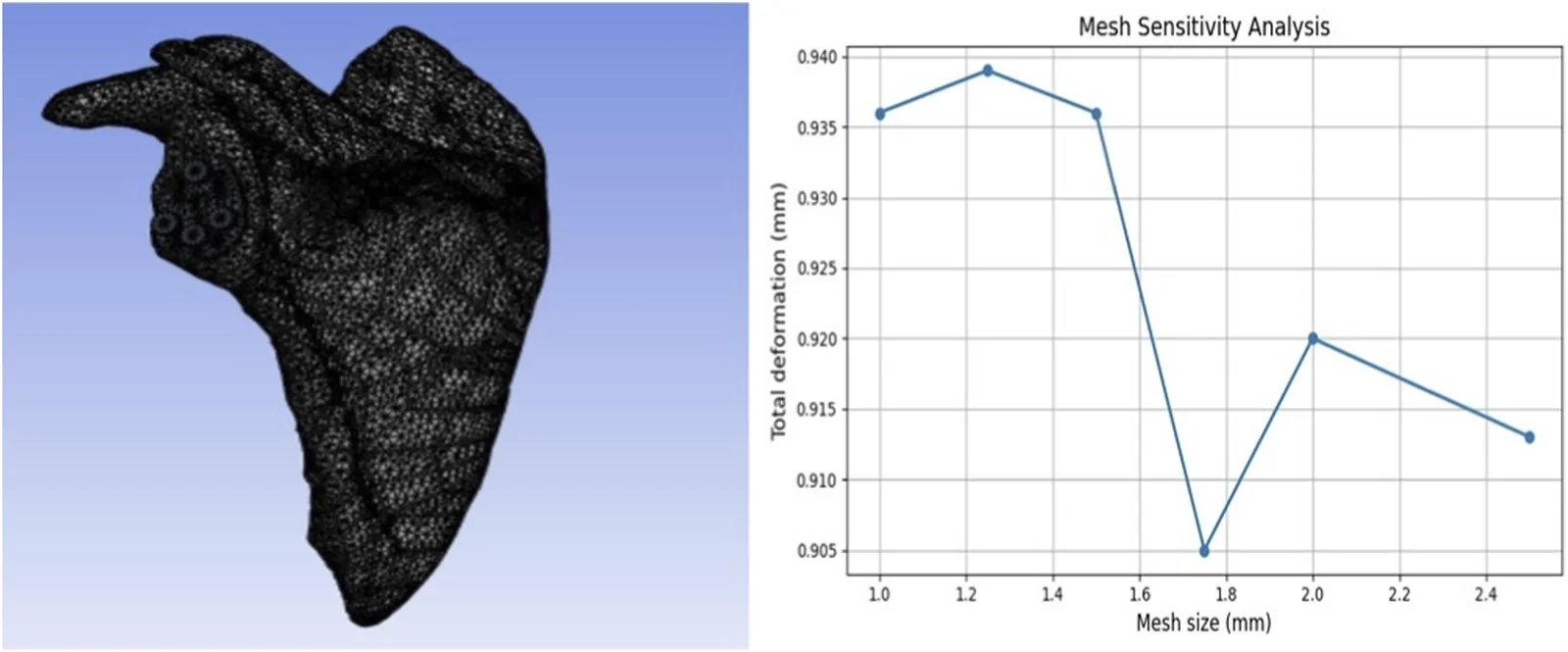
Meshed glenoid-scapula assembly and mesh sensitivity graph.

### 2.6 Contact definitions, loading and boundary conditions

Contact interactions were defined in Ansys Workbench, and a frictional contact with the coefficient of friction of µ = 0.3 was applied between the glenoid surface and the base of the baseplate. Frictional contact was applied at the implant-bone junction so that micromotion could be determined with frictional resistance between the baseplate and scapula. Contact pressure, penetration, gap and the sliding distance were measured for all simulations. Maximum sliding distance was interpreted as micromotion, which is a significant indicator of primary stability and fixation. Gap and penetration were determined to evaluate the local separation or compression at the junction, while contact pressure was found to determine the load transfer between the scapula and the glenoid baseplate.

The loading and boundary conditions were defined in Ansys Workbench to assess the primary stability of the scapula-screw-baseplate assembly under abduction loading. The restraint was fixed at the medial border of the scapula to restrict rigid body motion and create a supportive effect on the nearby anatomical attachments, as shown in Figure 7. The models were subjected to three abduction loading conditions, 0°, 30°, and 60˚respectively. For each abduction angle, the 750 N load was resolved into X, Y, and Z components and applied to the glenoid baseplate, showing that the load transfers from the glenosphere to the scapula-baseplate assembly during reverse shoulder arthroplasty, as listed in Table 5.

**FIGURE 7:**
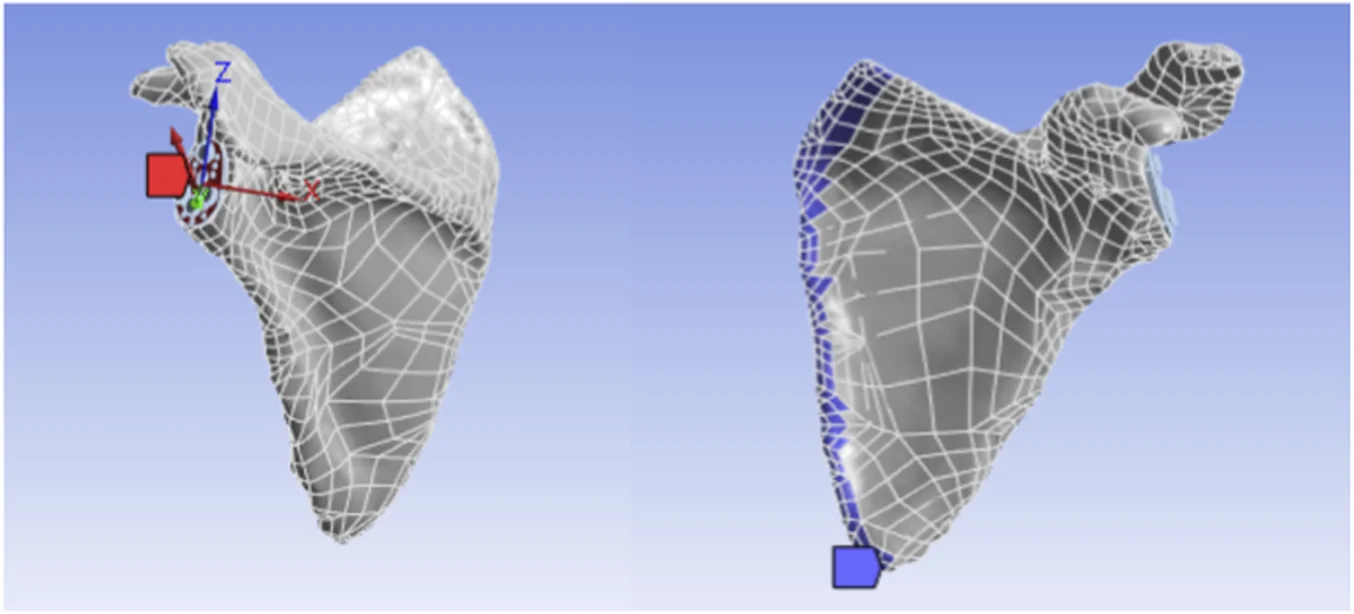
Loading and boundary conditions of the baseplate-bone construct.

**TABLE 5:** Resolved force components for different abduction angles.

Abduction angle	Fx (N)	Fy (N)	Fz (N)	Resultant force (N)
0°	0	0	750	750
30°	−375	0	649.5	750
60°	−649.5	0	375	750

### 2.7 Post-processing and outcome measures

The mechanical outcome of each assembly was evaluated during post-processing. Micromotion was determined using the maximum sliding distance. Maximum contact pressure at the baseplate - scapula junction was noted to determine the local load-transfer response. Von Mises stress was evaluated for the scapula and the baseplate. Additionally, the length of supporting bone stock (LSBS) was calculated for all the screws using Ansys SpaceClaim. These measurements denote the bone stock available along the screw paths adopted in the models. The contact area between the implant and glenoid surfaces was extracted from the contact tool in Ansys Mechanical.

## 3 Results

### 3.1 Baseplate stress response

The preliminary screening of pear-shaped baseplates with circular, oval, and elliptical hole patterns was carried out, and the corresponding von Mises stress response was evaluated, as shown in Figure 8. The Von Mises stress for circular, oval and elliptical baseplates ranged between 97 MPa and 579 MPa, 123 MPa–741 MPa, 258 MPa–1,552 MPa, respectively. The von Mises stress of baseplates was also screened based on Ti-6Al-4V and 316LSS for all three patterns.

**FIGURE 8:**
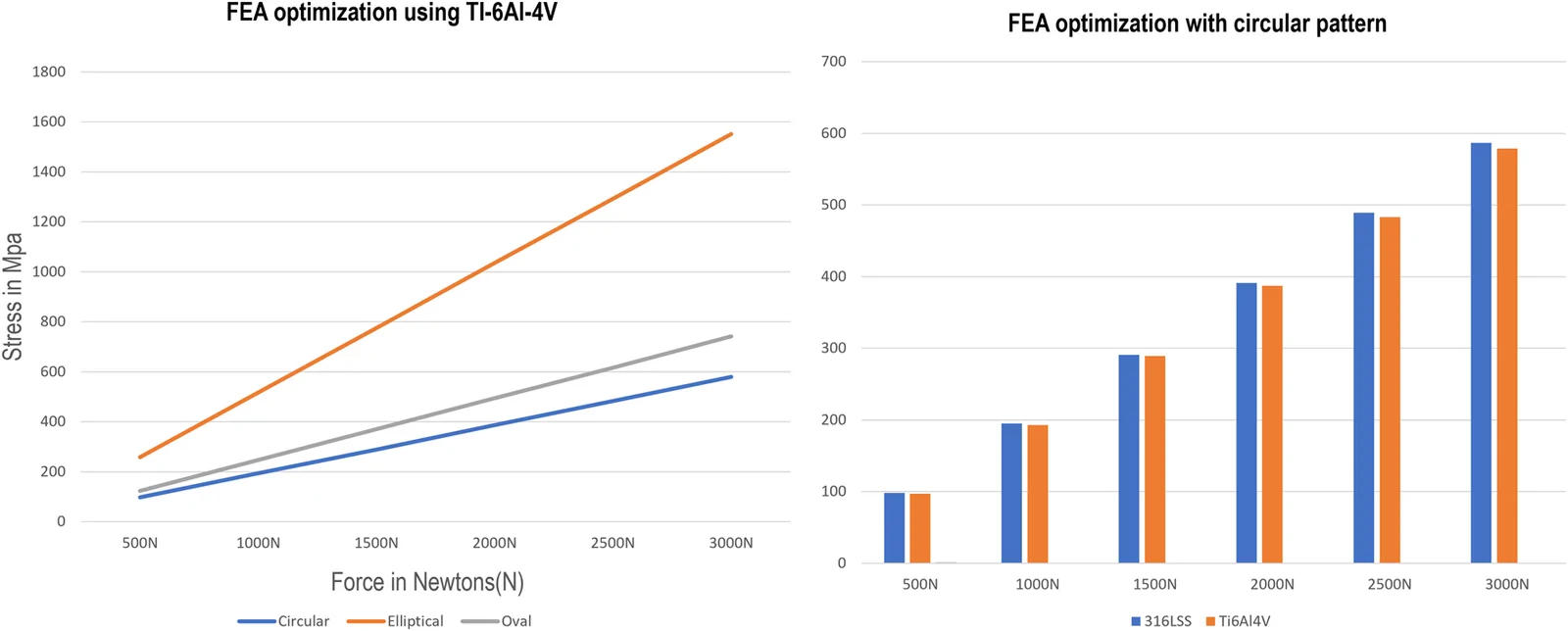
Von Mises stress response of the baseplates based on hole patterns and materials.

The results showed the baseplate with a circular pattern as the most effective pattern for stress distribution when compared to the elliptical and oval designs. The oval and elliptical patterns showed higher stress concentration around the internal pattern when compared to the circular pattern. Also, Ti-6Al-4V showed better stress response than 316LSS. Consequently, the baseplate with circular hole pattern and Ti-6Al-4V was selected for subsequent analysis.

### 3.2 Micromotion of the scapula-baseplate assembly

Micromotion was determined from the maximum sliding distance at the baseplate-bone interface. The sliding distance ranged from 37 µm to 61 μm, and all values remained below the 150 µm threshold, commonly used for primary fixation stability. The 20 mm baseplate produced the lowest micromotion on the 20 mm glenoid with a constant value of 37 µm for all the loading conditions. The 22 mm implant indicated uniform behaviour between 41 μm and 47 μm, and the 25 mm baseplate on the 22 mm glenoid experienced a constant value of 40 μm for all the loading conditions. These results indicate that the 20 mm baseplate was promising for the 20 mm glenoid. The 25 mm baseplate showed low micromotion on the 22 mm glenoid, and the 22 mm baseplate performed the most consistent or stable response across all the loading scenarios. Figure 9 represents the graph of micromotion, bone stress, contact stress and implant stress results. The results table based on 0°, 30°, and 60° loading conditions is provided in the supplementary file.

**FIGURE 9:**
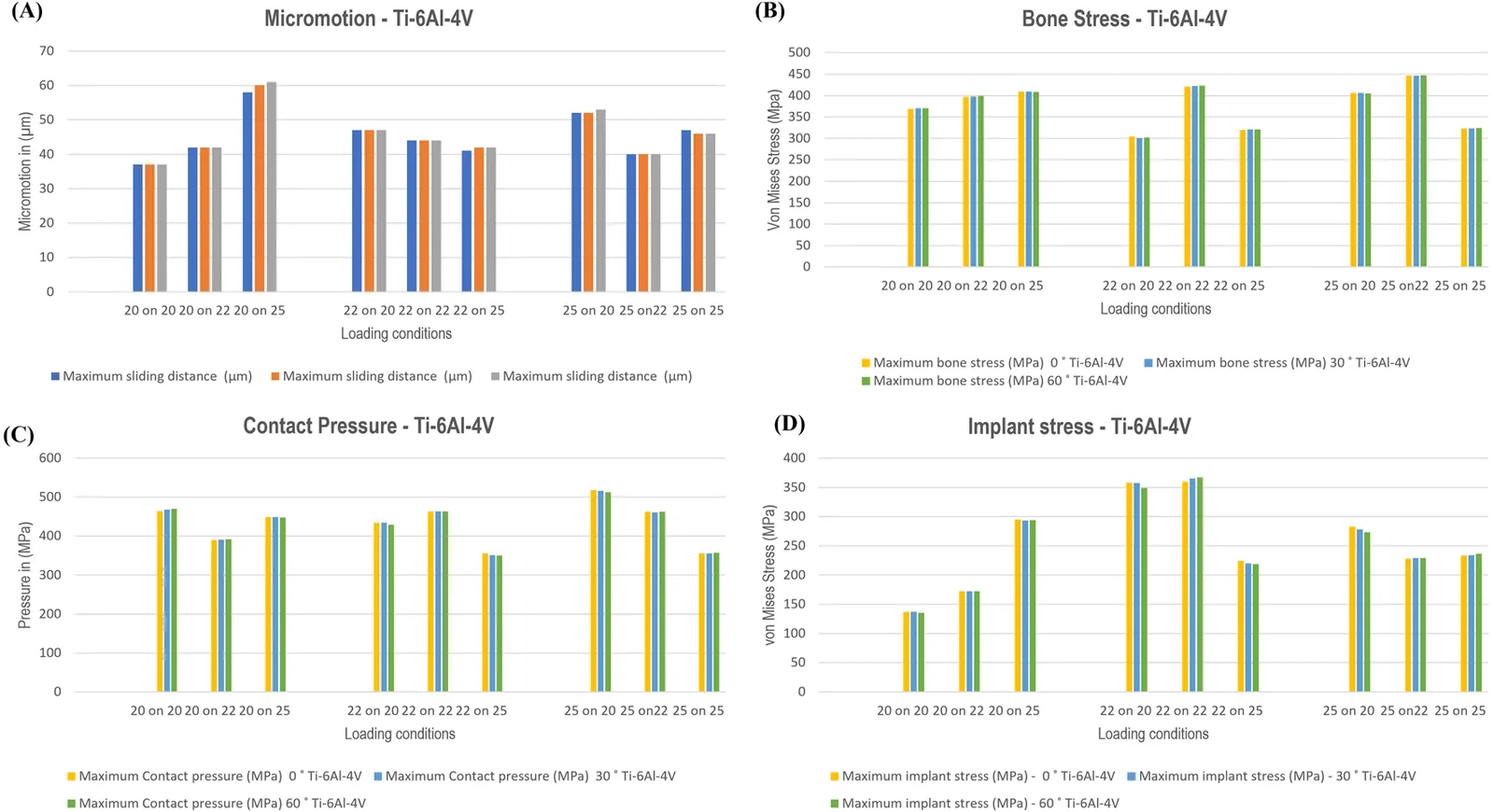
**(A)** Micromotion response, **(B)** bone stress, **(C)** contact pressure, **(D)** implant stress.

### 3.3 Scapula-baseplate contact response

Maximum contact pressure at the interface was between 350 MPa and 518 MPa throughout all baseplates and loading conditions. The contact pressure for 20 mm, 22 mm and 25 mm baseplates was found to be between 390 MPa and 470 MPa, 350 MPa and 463 MPa, and 355 MPa–518 MPa, respectively. 20 mm indicated constant pressure, 22 mm demonstrated variations based on the glenoid dimensions, and 25 mm showed some large variations when mounted on a 20 mm glenoid.

These results suggest that placing a large baseplate on a small glenoid enhanced localised interface compression. Even though the contact pressure was within the low-pressure range, local peak pressures were seen at contact regions. Hence, maximum contact pressure was viewed or interpreted as pressure at a localised region instead of consistent pressure throughout the surface of the baseplate. On the whole, the 20 mm baseplate produced uniform contact pressure, the 22 mm baseplate indicated a steady and controlled behaviour, especially with large glenoid dimensions, and the 25 mm baseplate experienced more sensitivity when a small glenoid model was analysed. The maximum penetration was found to be between 54 μm and 78 µm for all the models. The highest penetration was observed while the 25 mm baseplate was seated on a 20 mm glenoid, showing a greater localised compression. The lowest penetration was seen in the 22 mm baseplate on the 25 mm glenoid, indicating a regulated contact behaviour.

### 3.4 Scapular bone stress

Scapular bone stress was assessed using von Mises stress in the bone surrounding the baseplate and screws. Throughout all the models, the average scapular bone stress varied between 4.5 and 10.6 MPa. The highest average scapular stress was found in the 22–22 configuration at 60° loading. Maximum bone stress was found to extend between 301 MPa and 447 MPa throughout all the models and loading conditions. The lowest maximum bone stress was seen in the 22 on 20 model, and the highest was seen in the 25 on 22 model, as seen in Figure 10.

**FIGURE 10:**
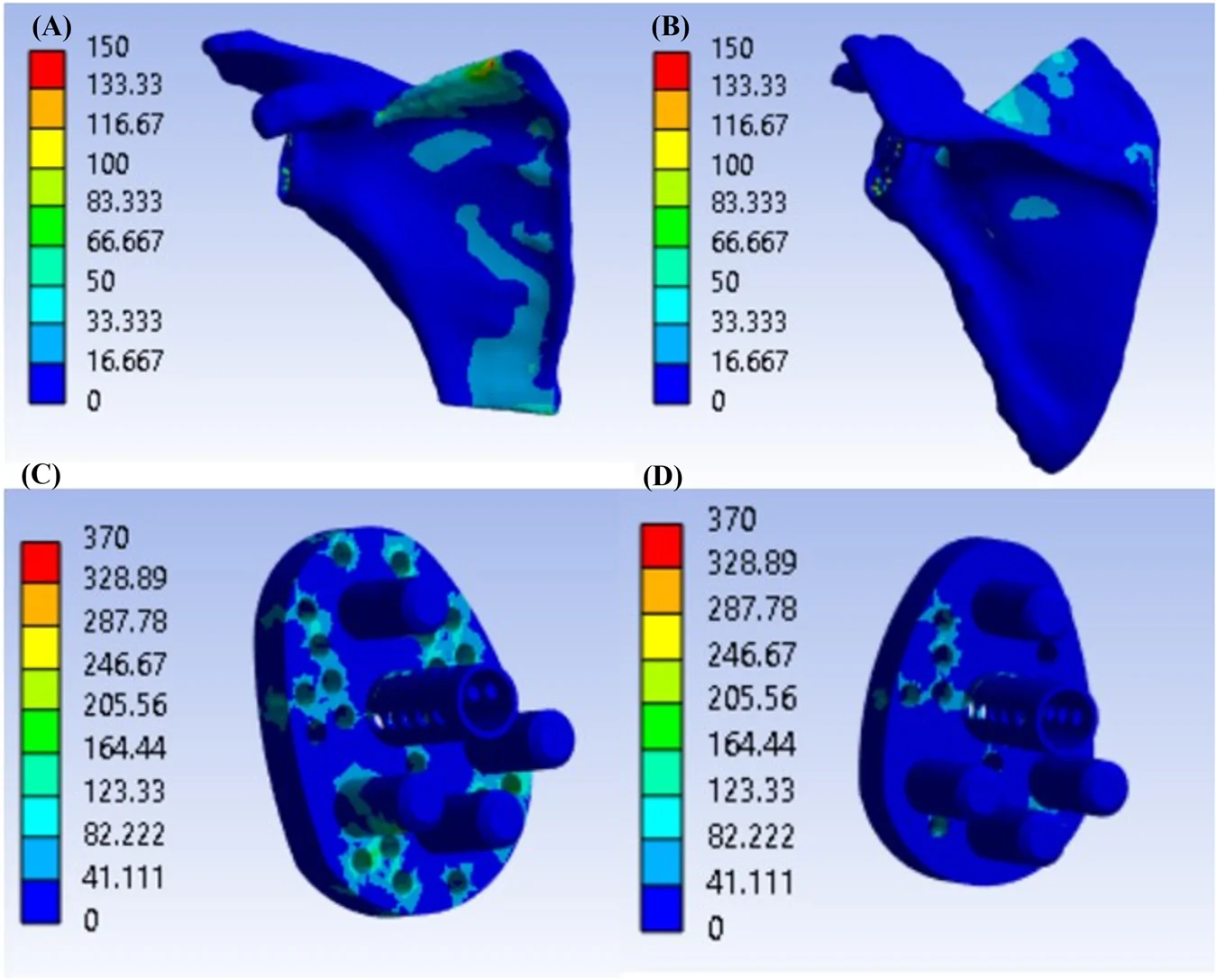
Representative von Mises stress for the models showing highest to lowest bone stress and implant stress. **(A)** Scapular bone stress in 25 on 22 at 60°, **(B)** scapular bone stress in 22 on 20 at 0°, **(C)** implant stress in 22 on 22 at 60°, **(D)** implant stress in 20 on 20 at 0°.

### 3.5 Implant stress

The implant stress was determined using von Mises stress, and it was found to range from 135 MPa to 367 MPa across all the loading conditions. As seen in Figure 10, the 20 mm baseplate demonstrated low stress, whereas the high implant stress was found on a 22 mm baseplate on a 22 mm glenoid, and 25 mm on 25 mm showed much lower stress for all the loading angles, indicating that the implant stress response varied based on the baseplate–glenoid size combination. On the whole, the baseplate stress was below the yield strength of Ti6Al4V, showing that the baseplate and screw construct was within the elastic limit across the loading angles.

### 3.6 Length of supporting bone stock and scapula–baseplate contact area

LSBS (Length of supporting bone stock) varied for different screw positions and baseplate-implant models, as shown in Table 6. The inferior screw involved a large portion of the bone stock, with values between 7.70 mm and 10.50 mm. The superior screw experienced increased bone support with values ranging between 4.10 mm and 10.50 mm.

**TABLE 6:** LSBS for various screw positions and contact area.

Case (baseplate on glenoid)	Anterior screw LSBS (mm)	Posterior screw LSBS (mm)	Superior screw LSBS (mm)	Inferior screw LSBS (mm)	Contact area (mm^2^)
20 on 20	4.14	3.97	7.45	8.8	193
20 on 22	3.3	3.6	8.8	10	164
20 on 25	4.3	3.34	6.6	7.7	188
22 on 20	3.3	2	8.3	10.5	198
22 on 22	2.3	2.5	10.5	8.8	186
22 on 25	3.5	2.6	4.1	8.2	208
25 on 20	4.58	3.1	8.3	8.7	255
25 on 22	6.2	3.86	9.06	8.6	248
25 on 25	2.2	2.4	4.3	8.8	258

On the contrary, anterior and posterior screws showed less bone support distributed between 2.20 and 6.20 mm and 2–3.97 mm, respectively. The maximum recorded LSBS for the inferior screw was seen in the 22 on 20 assembly, and the maximum value for the superior screw was observed on the 22 on 22 configuration.

The contact area between the glenoid baseplate and the glenoid revealed a size-dependent pattern as seen in Table 6. The contact area for 20 mm, 22 mm and 25 mm baseplates ranged between 164 and 193 mm^2^, 186 and 208 mm^2^, and 248 and 258 mm^2^, respectively. The 25 mm provided the greatest contact area for all the glenoid geometries, with the highest contact area observed for a 25 mm baseplate on a 25 mm glenoid model.

## 4 Discussion

Micromotion has been considered a significant measure of primary stability and fixation in studies on the biomechanics of reverse shoulder arthroplasty. In our study, the maximum sliding distance was observed between 37 μm and 61 µm for all the baseplate–scapula assemblies and loading conditions (Figure 9) (Bonnevialle et al., 2020; Formaini et al., 2015; Torkan et al., 2022; Parr et al., 2022). The measured values were below 150 μm, the threshold for bone ingrowth and osteointegration, suggesting that the baseplates provide sufficient primary stability under the loading conditions.

Lung et al. showed that baseplate micromotion depends on bone density, peripheral screw length and central peg length. It also showed that the baseplate micromotion was greater when bone density was reduced and when shorter screws and a shorter central peg (Lung et al., 2019).

The 20 mm baseplate revealed the lowest sliding distance of 37 μm**,** supporting the stability of a small baseplate for a smaller glenoid. Likewise, studies have proven that anatomically similar baseplates improved fixation and stability in small glenoids (Chae et al., 2014; Kim and Chae, 2026). 22mm baseplate indicated consistent micromotion response with values between 41 μm and 47 µm for all the models and loading conditions. This suggests that the 22 mm baseplate contributed a balanced response across the wider anatomical range for Indian glenoids.

The changes in total deformation across all models indicate that overall displacement depended on the loading scenarios and on the assembly and geometric configuration of the baseplate and the glenoid. The elevated deformation was observed at larger loading angles and suggested a higher mechanical demand on the model. Nevertheless, total deformation indicates the global outcome of the construct and should be viewed as a supporting result.

Noticeable changes in contact pressure were evident at the scapula-baseplate contact region for different models (Figure 9). Diaz et al. showed that greater compression at the baseplate–bone interface was linked with reduced micromotion, indicating that interface compression contributes to primary baseplate stability. This confirms the present observation that differences in contact response among the glenoid–baseplate models were accompanied by differences in micromotion (Diaz et al., 2024). These differences were primarily due to the load transfer pattern from the implant to the scapula based on the geometrical configurations between them. However, the peak contact pressure should be interpreted with caution as it represents the localised regions of high pressure rather than the mechanical outcome of the bone-implant interface. A greater peak value does not conclusively imply an undesirable fixation outcome if the enhanced pressure is localised to a particular region. Hence, the scapular stress patterns were also considered with the pressure findings to understand the interface behaviour.

The bone stress indicated that it changed with baseplate-glenoid size. Studies on FEA and biomechanics have demonstrated that bone–implant stress is influenced by glenoid support, fixation and implant design conditions (Bonnevialle et al., 2020; Sun et al., 2023; Yang et al., 2013). Von Mises stress for cortical bone determined by Bonnevialle et al. (2020) varied from 1.8MPa to 7 MPa and for the trabecular bone it was found to be between 0.6 MPa and 1.6 MPa when a combined loading of 756 N was applied.

In this work, the average scapular von Mises bone stress was found to be between 4.5 MPa and 10 MPa when subjected to a similar 750 N load (Figure 10). Some of these differences can be due to changes in scapular models, material properties defined, implant design and loading conditions. However, the maximum scapular stress lies between 301 MPa and 447 MPa. These values are primarily the localised peak von Mises stress around the glenoid region, baseplate footprint and screw insertion site. These differences between the maximum and average scapular stress indicate that the high von Mises stresses are concentrated to localized regions of the scapula and not across the entire bone.

Sun et al. indicated that reduced glenoid support produced small changes in stress when adequate screw fixation was maintained, while loss of screw fixation along with low bone support increased maximum stress and micromotion (Sun et al., 2023). These findings emphasise that the mechanical response of the glenoid–baseplate assembly depends on the available supporting bone and fixation, consistent with the variation in scapular stress observed among the different glenoid–baseplate combinations in the present study.

In a study by Kim and Chae (2026), a 29 mm baseplate developed higher bone stress than a 25 mm baseplate, with maximum stress concentrated around the inferior screw. Also, it is reported that the 25 mm baseplate enhanced the bone-screw contact region and bone stock for screws in small glenoids. Similarly, in our study, the lowest scapular stress was determined for the 22 mm baseplate when placed on the 20 mm glenoid and the highest stress for the 25 mm baseplate on the 22 mm glenoid. These investigations demonstrate that the scapular bone stress was dependent on the baseplate dimensions and the glenoid geometry rather than the baseplate diameter alone.

Chebli et al. (2008) demonstrated that fixation of the baseplate on the glenoid is dependent on the bone density, screw configuration and the direction of loading. Zhang et al. (2020) and other studies (Rojas et al., 2019; Schell et al., 2023) reported that stress-shielding-induced behaviour can lower bone density, which is one of the crucial indicators of aseptic loosening of the baseplate after RTSA. In our study, internal patterns in the form of holes were designed to reduce the effect of stress shielding by altering implant rigidity and rendering a favourable bone transfer. However, this is only a structural analysis; hence, the results are interpreted as a basic investigation of a changed load transmission instead of an explicit proof of stress shielding.

The implant von Mises stress determined for all models was below the yield strength of Ti-6Al-4V under all loading conditions (Figure 10). Yang et al. revealed through FEA that the stress generated within the glenoid baseplate is sensitive to the implant geometry and position, with changes in glenosphere placement and lateral offset altering stress at the glenoid–baseplate interface (Yang et al., 2013). Although these design variables differ from the baseplate dimensions evaluated in the present study, their investigations similarly indicate that implant stress is configuration-dependent rather than governed by a single geometric parameter.

Kim and Chae (2026) reported that the baseplate size should match the glenoid geometry for a favourable fixation. It was reported that a 25 mm baseplate resulted in more supportive fixation than a 29 mm baseplate, with less micromotion, bone stress, together with greater bone–screw support. Clinical investigations by Athwal and Faber (2016) indicated that small-sized implants were advantageous over the broader size for patients with a smaller glenoid. It has been reported that a 25 mm baseplate was selected when the standard baseplate was found to be disproportionate for the available glenoid width and did not result in any failure or loosening in a short-term follow-up. Limited studies remain regarding the assessment of the baseplate diameter and von Mises stress of the implant-baseplate assembly. Concerning the implant stress, the 20 mm baseplate produced a promising outcome on 20 mm and 22 mm glenoid models; however, the 22 mm baseplate showed better support for the 25 mm glenoid. Even though smaller baseplates were more suitable for the Indian geometry and revealed less stress when compared with the wider baseplate diameters, it did not result in less implant stress uniformly. This indicates that the implant stress is not only dependent upon the dimensions of the baseplate but also on the dimensional match between the glenoid and the baseplate.

The LSBS (length of supporting bone stock) changed based on the implant-bone and screw position (Table 6). The superior and inferior screws involved a better portion of supporting bone than the anterior and posterior screws. Roche et al. demonstrated that long peripheral screws substantially reduced baseplate displacement compared with shorter screws under cycling, showing the significance of the available bone length along screw trajectory for stable fixation (Roche et al., 2019). This supports the implication of the greater supporting bone stock seen along the superior and inferior screw trajectories in the present study. Similarly, Hart et al. reported greater supporting bone portion for the superior and inferior screws than anterior and posterior screws (Hart et al., 2013). Kim and Chae revealed that LSBS is governed by the geometrical match between the baseplate and the glenoid, where the small baseplate resulted in a larger supporting bone stock in small glenoids. While studies by Kim and Chae (2026) involved inserting screws through predefined angle positions, our study did not incorporate this optimisation; rather, the findings denote the bone support along the screw paths in a standard manner. In this work, a wider baseplate diameter alone did not result in a consistent change in LSBS throughout all screw positions. Nevertheless, the level of the screw support changed with respect to glenoid geometry and the implant dimensions. Though LSBS cannot evaluate implant stability, sufficient bone stock along the screw path can enhance screw anchorage and facilitate better stability.

The 25 mm baseplate also provided the greatest baseplate–glenoid contact area across the representative models (Table 6). Although the 25 mm matched model resulted in largest contact area, it did not produce low micromotion, showing that total interface area alone did not determine the initial fixation. Primary stability also depended on interface load, contact-pressure distribution and the supporting bone available along the screw trajectories. Hence, additional peripheral contact did not provide a corresponding improvement in resistance to interface motion. Bobko et al. reported that rotational malposition significantly reduced implant–bone contact area, emphasizing the importance of accurate baseplate seating in maintaining interface contact.

Other strategies have been proposed to address various challenges in glenoid baseplate design related to glenoid deformity, bone deficiency and fixation requirements. Lateralization strategies can change joint biomechanics and reduce scapular notching (Baumgarten and Max, 2024; King et al., 2023). Augmented and patient-specific baseplates are primarily used to manage glenoid deformity or bone deficiency (Levin et al., 2022; Colasanti et al., 2023; Rashid et al., 2023; Moran et al., 2024; Bodendorfer et al., 2021). On the contrary, the present approach focuses on dimensional compatibility between the baseplate and the available glenoid geometry. These strategies address different aspects of implant selection and may be complementary when dimensional mismatch coexists with glenoid deformity or bone loss.

These results demonstrate that the size match between the baseplate and the glenoid geometry drives the baseplate’s primary fixation. The 20 mm baseplate resulted in minimum micromotion from the smallest glenoid model, and the 22 mm baseplate produced a uniform behaviour throughout all the models with limited changes in relative motion. This implies that 20 mm and 22 mm baseplates can be appropriate for small glenoid dimensions of Indian models. LSBS observations reveal that 20 mm and 22 mm baseplates retained adequate bone stock along the superior and inferior screw positions when compared to the 25 mm baseplate. Scapular stress, implant stress, total deformation and contact pressure were found to vary for different implant-glenoid models, indicating that a larger baseplate does not inevitably ensure improved primary stability. 22 mm baseplate demonstrated desirable contact pressure and scapular stress for all the loading conditions. Nevertheless, it suggested that baseplate selection was based on the baseplate–glenoid configuration rather than the size.

The present study was limited to representative glenoid dimensions within a standardised morphology, and different morphological classes were not evaluated separately. Inter-and intra-observer reliability were not assessed. Variations in glenoid version, inclination, pathological bone loss and bone density were not evaluated as independent variables, although these factors can influence baseplate seating, supporting bone stock and fixation stability. The present study was specifically designed to evaluate the influence of baseplate-glenoid dimensional compatibility on primary stability. Also, the analysis was restricted to the use of pear-shaped baseplates and did not evaluate variations in screw trajectory, fatigue behaviour, stress shielding or osseointegration. Future studies should incorporate classified glenoid morphologies and additional anatomical and bone-quality variables. As the findings were obtained through finite element simulations, further cadaveric and clinical validation is required.

## 5 Conclusion

The evaluated biomechanical outcomes indicate that primary fixation depended more on baseplate–glenoid dimensional compatibility than on increasing baseplate size alone. These findings support morphometry-guided baseplate selection, with smaller and intermediate baseplates demonstrating favourable primary fixation for smaller glenoid dimensions in the evaluated Indian glenoid models.

## Data Availability

The original contributions presented in the study are included in the article/Supplementary Material, further inquiries can be directed to the corresponding author.
