# Supplementary material for: Morphometry-based design and finite element assessment of primary stability in glenoid baseplate fixation for reverse shoulder arthroplasty for the Indian population
Source: Front Bioeng Biotechnol. 2026 Oct 5;14:1927447. doi: 10.3389/fbioe.2026.1927447 (PMC13677933; doi:10.3389/fbioe.2026.1927447)
Supplement: Supplementary file 1 [file Table1.docx]

**Complete angle-wise biomechanical outcomes across 0°, loading condition.**

| **Case**  **(baseplate on glenoid) (mm)** | **Micromotion**  **Maximum sliding distance (µm)** | **Max penetration (µm)** | **Max contact pressure (MPa)** | **Maximum**  **bone stress** | **Average bone stress (MPa)** | **Peak implant stress (MPa)** |
| --- | --- | --- | --- | --- | --- | --- |
| 20 on 20 | 37 | 66 | 464 | 369 | 4.7 | 137 |
| 20 on 22 | 42 | 62 | 390 | 397 | 4.5 | 172 |
| 20 on 25 | 58 | 72 | 449 | 409 | 6.9 | 295 |
| 22 on 20 | 47 | 68 | 434 | 304 | 6.1 | 358 |
| 22 on 22 | 44 | 72 | 463 | 421 | 5.4 | 360 |
| 22 on 25 | 41 | 55 | 355 | 320 | 6.2 | 224 |
| 25 on 20 | 52 | 78 | 518 | 406 | 5.3 | 283 |
| 25 on 22 | 40 | 74 | 462 | 446 | 5.1 | 228 |
| 25 on 25 | 47 | 57 | 355 | 323 | 6.6 | 233 |

**Complete angle-wise biomechanical outcomes across 30°, loading condition.**

| **Case**  **(baseplate on glenoid) (mm)** | **Micromotion**  **Maximum sliding distance (µm)** | **Max penetration (µm)** | **Max contact pressure (MPa)** | **Maximum**  **bone stress** | **Average bone stress (MPa)** | **Peak implant stress (MPa)** |
| --- | --- | --- | --- | --- | --- | --- |
| 20 on 20 | 37 | 67 | 468 | 370 | 5.7 | 137 |
| 20 on 22 | 42 | 62 | 391 | 398 | 7.4 | 172 |
| 20 on 25 | 60 | 72 | 449 | 409 | 9.2 | 293 |
| 22 on 20 | 47 | 68 | 434 | 301 | 6.08 | 357 |
| 22 on 22 | 44 | 72 | 463 | 422 | 8.8 | 365 |
| 22 on 25 | 42 | 54 | 351 | 321 | 6.09 | 220 |
| 25 on 20 | 52 | 77 | 516 | 406 | 7.9 | 278 |
| 25 on 22 | 40 | 74 | 461 | 446 | 8.04 | 229 |
| 25 on 25 | 46 | 57 | 355 | 323 | 8.8 | 234 |

**Complete angle-wise biomechanical outcomes across 60°, loading condition.**

| **Case**  **(baseplate on glenoid) (mm)** | **Micromotion**  **Maximum sliding distance (µm)** | **Max penetration (µm)** | **Max contact pressure (MPa)** | **Maximum**  **bone stress** | **Average bone stress (MPa)** | **Peak implant stress (MPa)** |
| --- | --- | --- | --- | --- | --- | --- |
| 20 on 20 | 37 | 67 | 470 | 370 | 5.6 | 135 |
| 20 on 22 | 42 | 62 | 392 | 399 | 9.08 | 172 |
| 20 on 25 | 61 | 72 | 448 | 408 | 9.8 | 294 |
| 22 on 20 | 47 | 68 | 429 | 302 | 6.2 | 349 |
| 22 on 22 | 44 | 72 | 463 | 423 | 10.6 | 367 |
| 22 on 25 | 42 | 54 | 350 | 321 | 9.3 | 219 |
| 25 on 20 | 53 | 77 | 512 | 405 | 9.3 | 273 |
| 25 on 22 | 40 | 74 | 462 | 447 | 9.7 | 229 |
| 25 on 25 | 46 | 57 | 357 | 324 | 9.2 | 236 |
